# Draft Genome Sequence of Moorella sp. Strain Hama-1, a Novel Acetogenic Bacterium Isolated from a Thermophilic Digestion Reactor

**DOI:** 10.1128/genomeA.00517-18

**Published:** 2018-06-14

**Authors:** Jun Harada, Takeshi Yamada, Surya Giri, Masako Hamada, Masaru K. Nobu, Takashi Narihiro, Hideto Tsuji, Hiroyuki Daimon

**Affiliations:** a Department of Environmental and Life Sciences, Toyohashi University of Technology, Toyohashi, Aichi, Japan; b Bioproduction Research Institute, National Institute of Advanced Industrial Science and Technology (AIST), Tsukuba, Ibaraki, Japan; c Institute for Global Network Innovation in Technology Education, Toyohashi University of Technology, Toyohashi, Aichi, Japan

## Abstract

Moorella sp. strain Hama-1 was isolated from a thermophilic anaerobic digestion reactor treating poly(l-lactic acid). The strain is a thermophilic acetogen capable of lactate oxidation under anaerobic conditions. Here, we report the draft genome sequence of strain Hama-1, comprising 3.27 Mb in 48 contigs, with a G+C content of 56.6%.

## GENOME ANNOUNCEMENT

The genus Moorella currently contains seven species of strictly anaerobic thermophilic bacteria (1–6). This genus includes Moorella thermoacetica, a representative of acetogens (7). Although some species of this genus can utilize the Wood-Ljungdahl pathway for CO and CO_2_ fixation and energy conservation, which is a characteristic feature of acetogens (7–10), the rest of the species of this genus cannot utilize H_2_/CO_2_ as carbon and energy sources (2–5). However, the reasons for the differences in such core physiological characteristics among the Moorella species are still unclear. To gain more insight into the ecophysiology of Moorella species, a novel strain, Hama-1, was isolated from a thermophilic digestion reactor treating poly(l-lactic acid). Although strain Hama-1 shares 99.6% 16S rRNA gene sequence similarity with Moorella perchloratireducens strain An10^T^ (4), there are many phenotypic differences between the two strains, e.g., temperature range for growth, NaCl requirements, and substrate utilization. In particular, only the strain Hama-1 could utilize H_2_/CO_2_ and lactate as carbon and energy sources and could produce acetate as a sole end product. To clarify these phenotypic differences among the Moorella organisms, a draft genome sequence for strain Hama-1 was determined.

DNA from pure culture of strain Hama-1 was extracted according to a previous method (11), with the addition of lysozyme and phenol-chloroform treatment. Whole-genome shotgun sequencing was conducted using the Illumina MiSeq platform (San Diego, CA, USA) at the Bioengineering Lab. Co., Ltd. (Kanagawa, Japan). The paired-end library was constructed and sequenced, and paired-end reads (875,835 pairs) were obtained. Read sequences of ≤127 bp and their paired-read sequences were discarded after removing bases with a quality score of ≤Q20 using Sickle software version 1.33 (https://github.com/najoshi/sickle). *De novo* assembly of high-quality reads (805,026 pairs) was performed using SPAdes software version 3.10.1 (12). The assembled data resulted in 48 contigs (>1,000 bp), with 147.7-fold average coverage. Function prediction and annotation of the draft genome were performed using the Prokka pipeline version 1.12 (13).

The obtained draft genome sequence of strain Hama-1 comprised 3.27 Mb, with a G+C content of 56.6%. The genome harbored 3,132 protein-coding and 54 RNA-coding genes, including 2 rRNAs and 51 tRNAs. Genes encoding enzymes of the methyl and carbonyl branches of the Wood-Ljungdahl pathway that are conserved among known acetogens (7, 14) were identified. The draft genome also contained genes encoding enzymes involved in lactate oxidation (lactate dehydrogenase, pyruvate ferredoxin oxidoreductase, phosphotransacetylase, and acetate kinase). Further comparative genomic analysis will not only lead to a better understanding of the phenotypic differences between strain Hama-1 and other Moorella species and strains, including M. perchloratireducens strain An10^T^, but also will provide insight into the ecological niches of diverse Moorella species.

### Accession number(s).

The draft genome sequence has been deposited to the DDBJ/GenBank/EMBL under the accession number BFFN00000000. The version described in this paper is version BFFN01000000.
